# 9-(4-Fluoro­phen­yl)-3,3,6,6-tetra­methyl-10-*p*-tolyl-1,2,3,4,5,6,7,8,9,10-deca­hydroacridine-1,8-dione

**DOI:** 10.1107/S1600536808027256

**Published:** 2008-08-30

**Authors:** Ziqiang Tang, Changning Liu, Shanshan Wu, Wenjuan Hao

**Affiliations:** a School of Chemistry and Chemical Engineering, Xuzhou Institute of Technology, Xuzhou 221006, People’s Republic of China; b School of Chemistry and Chemical Engineering, Xuzhou Normal University, Xuzhou 221116, People’s Republic of China

## Abstract

The title compound, C_30_H_32_FNO_2_, was synthesized by the reaction of dimedone with 4-fluoro­benzaldehyde and *p*-toluidine in water. The dihydro­pyridine and both of the cyclo­hexenone rings are not planar and have flattened boat conformations. The dihedral angle between the planar aromatic rings is 15.33 (3)°. In the crystal structure, inter­molecular C—H⋯O hydrogen bonds link the mol­ecules into centrosymmetric dimers.

## Related literature

For general background, see: Wysocka-Skrzela & Ledochowski (1976 ▶); Nasim & Brychcy (1979 ▶); Thull & Testa, (1994 ▶); Reil *et al.* (1994 ▶); Mandi *et al.* (1994 ▶). For related literature, see: Tu *et al.* (2004 ▶). For ring puckering parameters, see: Cremer & Pople (1975 ▶). For bond-length data, see: Allen *et al.* (1987 ▶).
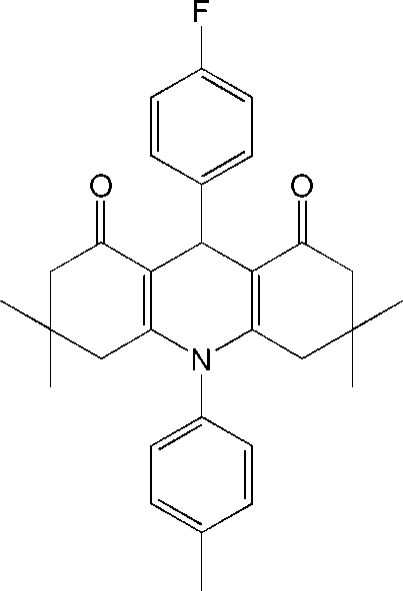


## Experimental

### 

#### Crystal data

C_30_H_32_FNO_2_
*M* *_r_* = 457.58
Monoclinic, 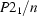

*a* = 15.1533 (15) Å
*b* = 10.9643 (12) Å
*c* = 16.1053 (15) Å
β = 102.317 (2)°
*V* = 2614.2 (5) Å^3^
*Z* = 4
Mo *K*α radiation
μ = 0.08 mm^−1^
*T* = 298 (2) K
0.37 × 0.25 × 0.21 mm

#### Data collection

Bruker SMART CCD area-detector diffractometer
Absorption correction: multi-scan (*SADABS*; Sheldrick, 1996 ▶) *T*_min_ = 0.972, *T*_max_ = 0.984
13238 measured reflections
4605 independent reflections
2277 reflections with *I* > 2σ(*I*)
*R*_int_ = 0.045

#### Refinement

*R*[*F* ^2^ > 2σ(*F* ^2^)] = 0.051
*wR*(*F* ^2^) = 0.152
*S* = 1.02
4605 reflections
307 parameters
H-atom parameters constrained
Δρ_max_ = 0.18 e Å^−3^
Δρ_min_ = −0.20 e Å^−3^

### 

Data collection: *SMART* (Bruker, 1997 ▶); cell refinement: *SAINT* (Bruker, 1997 ▶); data reduction: *SAINT*; program(s) used to solve structure: *SHELXS97* (Sheldrick, 2008 ▶); program(s) used to refine structure: *SHELXL97* (Sheldrick, 2008 ▶); molecular graphics: *SHELXTL* (Sheldrick, 2008 ▶); software used to prepare material for publication: *SHELXTL*.

## Supplementary Material

Crystal structure: contains datablocks global, I. DOI: 10.1107/S1600536808027256/hk2514sup1.cif

Structure factors: contains datablocks I. DOI: 10.1107/S1600536808027256/hk2514Isup2.hkl

Additional supplementary materials:  crystallographic information; 3D view; checkCIF report

## Figures and Tables

**Table 1:** Hydrogen-bond geometry (Å, °)

*D*—H⋯*A*	*D*—H	H⋯*A*	*D*⋯*A*	*D*—H⋯*A*
C15—H15⋯O1^i^	0.93	2.45	3.336 (3)	159

Symmetry code: (i) 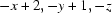.

**Fig. 1.**
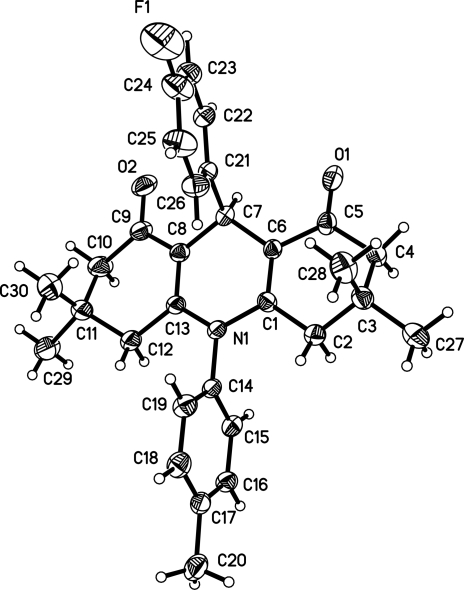
The molecular structure of the title molecule, with the atom-numbering scheme. Displacement ellipsoids are drawn at the 30% probability level.

**Fig. 2.**
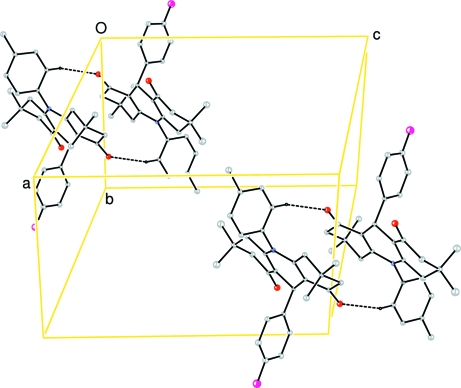
A partial packing diagram of the title compound. Hydrogen bonds are shown as dashed lines.

## supplementary crystallographic information

### Comment

Acridine derivatives containing 1,4-dihydropyridine unit belong to a special class of compounds not only because of their interesting chemical and physical properties but also due to their immense utility in pharmaceutical and dye industry, and they are well known atherapeutic agents (Wysocka-Skrzela & Ledochowski, 1976; Nasim & Brychcy, 1979; Thull & Testa, 1994; Reil *et al.*, 1994; Mandi *et al.*, 1994). We have reported the synthesis of *N*-hydroxylacridine derivatives, previously, (Tu *et al.*, 2004) and we report herein the structure of the title compound.

In the molecule of the title compound (Fig. 1), the bond lengths (Allen *et al.*,  1987) and angles are generally within normal ranges. Rings D (C14-C19) and E (C21-C26) are, of course, planar and they are oriented at a dihedral angle of D/E = 15.33 (3)°. Rings A (C1-C6), B (N1/C1/C6-C8/C13) and C (C8-C13) are not planar, having total puckering amplitudes, Q_T_, of 0.475 (3), 0.201 (2) and 0.448 (3) Å, respectively, and flattened boat conformations [φ = -56.70 (3)°, θ = 120.78 (3)°; φ = 172.92 (2)°, θ = 76.41 (3)° and φ = 170.16 (3)°, θ = 54.52 (3)°, respectively] (Cremer & Pople, 1975).

In the crystal structure, intermolecular C-H···O hydrogen bonds (Table 1) link the molecules into centrosymmetric dimers (Fig. 2), in which they may be effective in the stabilization of the structure.

### Experimental

The title compound was prepared by the reaction of dimedone (2 mmol) with 4-fluorobenzaldehyde (1 mmol) and *p*-toluidine in water (1 mmol) at 413 K under microwave irradiation (maximum power 140 W, initial power 120 W) for 12 min (yield; 89%). Single crystals suitable for X-ray analysis were obtained from an ethanol solution by slow evaporation.

### Refinement

H atoms were positioned geometrically, with C-H = 0.93, 0.98, 0.97 and 0.96 Å for aromatic, methine, methylene and methyl H, respectively, and constrained to ride on their parent atoms with U_iso_(H) = xU_eq_(C), where x = 1.5 for methyl H and x = 1.2 for all other H atoms.

### Crystal data

**Table:**

C_30_H_32_FNO_2_	*F*_000_ = 976
*M**_r_* = 457.58	*D*_x_ = 1.163 Mg m^−^^3^
Monoclinic, *P*2_1_/*n*	Melting point = 540–541 K
Hall symbol: -P 2yn	Mo *K*α radiation λ = 0.71073 Å
*a* = 15.1533 (15) Å	Cell parameters from 2294 reflections
*b* = 10.9643 (12) Å	θ = 2.3–2.3º
*c* = 16.1053 (15) Å	µ = 0.08 mm^−^^1^
β = 102.317 (2)º	*T* = 298 (2) K
*V* = 2614.2 (5) Å^3^	Block, pale yellow
*Z* = 4	0.37 × 0.25 × 0.21 mm

### Data collection

**Table:**

Bruker SMART CCD area-detector diffractometer	4605 independent reflections
Radiation source: fine-focus sealed tube	2277 reflections with *I* > 2σ(*I*)
Monochromator: graphite	*R*_int_ = 0.045
*T* = 298(2) K	θ_max_ = 25.0º
φ and ω scans	θ_min_ = 1.7º
Absorption correction: multi-scan(SADABS; Sheldrick, 1996)	*h* = −16→18
*T*_min_ = 0.972, *T*_max_ = 0.984	*k* = −13→12
13238 measured reflections	*l* = −13→19

### Refinement

**Table:**

Refinement on *F*^2^	Secondary atom site location: difference Fourier map
Least-squares matrix: full	Hydrogen site location: inferred from neighbouring sites
*R*[*F*^2^ > 2σ(*F*^2^)] = 0.052	H-atom parameters constrained
*wR*(*F*^2^) = 0.152	*w* = 1/[σ^2^(*F*_o_^2^) + (0.0429*P*)^2^ + 1.7217*P*] where *P* = (*F*_o_^2^ + 2*F*_c_^2^)/3
*S* = 1.02	(Δ/σ)_max_ < 0.001
4605 reflections	Δρ_max_ = 0.18 e Å^−^^3^
307 parameters	Δρ_min_ = −0.20 e Å^−^^3^
Primary atom site location: structure-invariant direct methods	Extinction correction: none

### Special details

**Table:**

Geometry. All e.s.d.'s (except the e.s.d. in the dihedral angle between two l.s. planes) are estimated using the full covariance matrix. The cell e.s.d.'s are taken into account individually in the estimation of e.s.d.'s in distances, angles and torsion angles; correlations between e.s.d.'s in cell parameters are only used when they are defined by crystal symmetry. An approximate (isotropic) treatment of cell e.s.d.'s is used for estimating e.s.d.'s involving l.s. planes.
Refinement. Refinement of *F*^2^ against ALL reflections. The weighted *R*-factor *wR* and goodness of fit *S* are based on *F*^2^, conventional *R*-factors *R* are based on *F*, with *F* set to zero for negative *F*^2^. The threshold expression of *F*^2^ > σ(*F*^2^) is used only for calculating *R*-factors(gt) *etc*. and is not relevant to the choice of reflections for refinement. *R*-factors based on *F*^2^ are statistically about twice as large as those based on *F*, and *R*- factors based on ALL data will be even larger.

### Fractional atomic coordinates and isotropic or equivalent isotropic displacement parameters (Å^2^)

**Table:**

	*x*	*y*	*z*	*U*_iso_*/*U*_eq_	
F1	0.73692 (17)	−0.0186 (2)	0.21288 (18)	0.1213 (10)	
N1	0.99513 (16)	0.5507 (2)	0.20652 (15)	0.0415 (6)	
O1	0.89407 (16)	0.3144 (2)	−0.03980 (14)	0.0634 (7)	
O2	1.13495 (16)	0.1657 (2)	0.20291 (16)	0.0695 (7)	
C1	0.93912 (19)	0.5309 (2)	0.12692 (19)	0.0389 (7)	
C2	0.8806 (2)	0.6360 (3)	0.08813 (19)	0.0485 (8)	
H2A	0.9169	0.6920	0.0628	0.058*	
H2B	0.8599	0.6794	0.1329	0.058*	
C3	0.7985 (2)	0.5977 (3)	0.0204 (2)	0.0507 (8)	
C4	0.8317 (2)	0.5133 (3)	−0.0417 (2)	0.0545 (9)	
H4A	0.7797	0.4807	−0.0814	0.065*	
H4B	0.8668	0.5606	−0.0740	0.065*	
C5	0.8883 (2)	0.4090 (3)	0.00008 (19)	0.0442 (8)	
C6	0.93853 (19)	0.4227 (2)	0.08715 (18)	0.0372 (7)	
C7	0.98875 (19)	0.3129 (2)	0.12966 (18)	0.0393 (7)	
H7	1.0187	0.2735	0.0885	0.047*	
C8	1.06046 (19)	0.3524 (2)	0.20419 (19)	0.0398 (7)	
C9	1.1334 (2)	0.2666 (3)	0.2352 (2)	0.0504 (8)	
C10	1.2098 (2)	0.3074 (3)	0.3049 (2)	0.0711 (11)	
H10A	1.2559	0.3447	0.2797	0.085*	
H10B	1.2364	0.2364	0.3366	0.085*	
C11	1.1820 (3)	0.3972 (3)	0.3660 (2)	0.0678 (11)	
C12	1.1319 (2)	0.5027 (3)	0.3152 (2)	0.0572 (9)	
H12A	1.1037	0.5516	0.3525	0.069*	
H12B	1.1753	0.5540	0.2954	0.069*	
C13	1.06083 (19)	0.4636 (2)	0.24020 (18)	0.0405 (7)	
C14	0.9980 (2)	0.6695 (2)	0.24664 (19)	0.0405 (7)	
C15	1.0480 (2)	0.7618 (3)	0.2215 (2)	0.0456 (8)	
H15	1.0790	0.7485	0.1783	0.055*	
C16	1.0518 (2)	0.8741 (3)	0.2606 (2)	0.0485 (8)	
H16	1.0857	0.9363	0.2435	0.058*	
C17	1.0058 (2)	0.8960 (3)	0.3249 (2)	0.0498 (8)	
C18	0.9566 (2)	0.8024 (3)	0.3491 (2)	0.0584 (9)	
H18	0.9258	0.8153	0.3925	0.070*	
C19	0.9519 (2)	0.6893 (3)	0.3101 (2)	0.0530 (9)	
H19	0.9177	0.6271	0.3268	0.064*	
C20	1.0093 (3)	1.0207 (3)	0.3652 (2)	0.0731 (11)	
H20A	0.9604	1.0696	0.3347	0.110*	
H20B	1.0657	1.0591	0.3633	0.110*	
H20C	1.0038	1.0126	0.4232	0.110*	
C21	0.9223 (2)	0.2213 (2)	0.15367 (18)	0.0410 (7)	
C22	0.9213 (2)	0.1009 (3)	0.1283 (2)	0.0535 (9)	
H22	0.9627	0.0739	0.0973	0.064*	
C23	0.8588 (3)	0.0196 (3)	0.1489 (2)	0.0692 (11)	
H23	0.8580	−0.0616	0.1321	0.083*	
C24	0.7990 (3)	0.0615 (4)	0.1941 (3)	0.0720 (11)	
C25	0.7985 (3)	0.1776 (4)	0.2209 (3)	0.0757 (12)	
H25	0.7572	0.2030	0.2525	0.091*	
C26	0.8606 (2)	0.2581 (3)	0.2007 (2)	0.0563 (9)	
H26	0.8609	0.3386	0.2191	0.068*	
C27	0.7548 (3)	0.7116 (3)	−0.0263 (2)	0.0761 (12)	
H27A	0.7360	0.7657	0.0135	0.114*	
H27B	0.7033	0.6881	−0.0691	0.114*	
H27C	0.7978	0.7524	−0.0526	0.114*	
C28	0.7295 (2)	0.5314 (3)	0.0606 (2)	0.0696 (11)	
H28A	0.7564	0.4592	0.0889	0.104*	
H28B	0.6781	0.5093	0.0172	0.104*	
H28C	0.7105	0.5842	0.1011	0.104*	
C29	1.2664 (3)	0.4479 (3)	0.4266 (3)	0.1137 (19)	
H29A	1.2483	0.4989	0.4684	0.171*	
H29B	1.3013	0.4948	0.3948	0.171*	
H29C	1.3022	0.3815	0.4544	0.171*	
C30	1.1203 (4)	0.3354 (4)	0.4167 (3)	0.1026 (16)	
H30A	1.0663	0.3081	0.3787	0.154*	
H30B	1.1048	0.3925	0.4565	0.154*	
H30C	1.1510	0.2668	0.4469	0.154*	

### Atomic displacement parameters (Å^2^)

**Table:**

	*U*^11^	*U*^22^	*U*^33^	*U*^12^	*U*^13^	*U*^23^
F1	0.0961 (19)	0.1090 (19)	0.155 (2)	−0.0497 (16)	0.0192 (17)	0.0503 (18)
N1	0.0426 (15)	0.0312 (13)	0.0459 (16)	0.0000 (11)	−0.0012 (12)	−0.0048 (11)
O1	0.0785 (18)	0.0585 (15)	0.0505 (15)	−0.0079 (13)	0.0076 (12)	−0.0145 (12)
O2	0.0646 (17)	0.0463 (14)	0.0919 (19)	0.0124 (12)	0.0039 (14)	−0.0141 (13)
C1	0.0361 (17)	0.0409 (17)	0.0382 (19)	−0.0049 (14)	0.0043 (14)	0.0037 (14)
C2	0.049 (2)	0.0423 (17)	0.051 (2)	0.0021 (15)	0.0023 (16)	−0.0008 (15)
C3	0.046 (2)	0.053 (2)	0.050 (2)	−0.0016 (17)	0.0020 (16)	0.0043 (16)
C4	0.057 (2)	0.062 (2)	0.041 (2)	−0.0095 (18)	0.0010 (16)	0.0063 (17)
C5	0.046 (2)	0.0477 (19)	0.0393 (19)	−0.0125 (16)	0.0105 (15)	−0.0029 (16)
C6	0.0376 (17)	0.0364 (16)	0.0368 (18)	−0.0066 (14)	0.0062 (14)	−0.0008 (13)
C7	0.0415 (18)	0.0359 (16)	0.0404 (18)	−0.0031 (14)	0.0087 (14)	−0.0067 (14)
C8	0.0363 (18)	0.0353 (16)	0.047 (2)	−0.0025 (14)	0.0062 (15)	−0.0010 (14)
C9	0.046 (2)	0.0369 (18)	0.065 (2)	−0.0009 (15)	0.0051 (17)	−0.0025 (16)
C10	0.061 (2)	0.050 (2)	0.089 (3)	0.0101 (19)	−0.015 (2)	−0.001 (2)
C11	0.081 (3)	0.046 (2)	0.062 (2)	0.004 (2)	−0.018 (2)	0.0009 (19)
C12	0.060 (2)	0.0403 (18)	0.061 (2)	−0.0004 (16)	−0.0105 (18)	−0.0054 (16)
C13	0.0396 (18)	0.0340 (16)	0.044 (2)	−0.0022 (14)	0.0012 (15)	−0.0003 (14)
C14	0.0408 (19)	0.0330 (16)	0.044 (2)	0.0009 (14)	0.0016 (15)	−0.0054 (14)
C15	0.0439 (19)	0.0419 (18)	0.050 (2)	−0.0034 (15)	0.0084 (15)	−0.0062 (15)
C16	0.049 (2)	0.0406 (18)	0.054 (2)	−0.0056 (15)	0.0059 (17)	0.0000 (15)
C17	0.052 (2)	0.0434 (19)	0.048 (2)	0.0073 (16)	−0.0028 (17)	−0.0074 (16)
C18	0.064 (2)	0.058 (2)	0.056 (2)	0.0031 (19)	0.0190 (18)	−0.0095 (18)
C19	0.057 (2)	0.0455 (19)	0.058 (2)	−0.0042 (17)	0.0164 (18)	−0.0023 (16)
C20	0.086 (3)	0.053 (2)	0.072 (3)	0.011 (2)	−0.001 (2)	−0.0208 (19)
C21	0.0423 (19)	0.0354 (16)	0.0423 (19)	−0.0061 (14)	0.0023 (15)	−0.0013 (14)
C22	0.061 (2)	0.0388 (18)	0.056 (2)	−0.0052 (17)	0.0011 (17)	−0.0027 (16)
C23	0.079 (3)	0.040 (2)	0.074 (3)	−0.018 (2)	−0.016 (2)	0.0088 (18)
C24	0.056 (3)	0.068 (3)	0.087 (3)	−0.020 (2)	0.004 (2)	0.030 (2)
C25	0.065 (3)	0.074 (3)	0.094 (3)	−0.002 (2)	0.030 (2)	0.022 (2)
C26	0.059 (2)	0.050 (2)	0.065 (2)	−0.0017 (17)	0.0231 (19)	0.0075 (17)
C27	0.070 (3)	0.070 (2)	0.076 (3)	0.012 (2)	−0.012 (2)	0.011 (2)
C28	0.047 (2)	0.091 (3)	0.069 (3)	−0.006 (2)	0.0102 (19)	0.002 (2)
C29	0.120 (4)	0.060 (3)	0.116 (4)	0.016 (3)	−0.074 (3)	−0.015 (3)
C30	0.169 (5)	0.073 (3)	0.060 (3)	−0.002 (3)	0.011 (3)	0.004 (2)

### Geometric parameters (Å, °)

**Table:**

F1—C24	1.367 (4)	C14—C15	1.376 (4)
N1—C1	1.395 (4)	C15—C16	1.379 (4)
N1—C13	1.403 (3)	C15—H15	0.9300
N1—C14	1.450 (3)	C16—C17	1.386 (4)
O1—C5	1.233 (3)	C16—H16	0.9300
O2—C9	1.225 (3)	C17—C18	1.373 (4)
C1—C6	1.347 (4)	C17—C20	1.510 (4)
C1—C2	1.506 (4)	C18—C19	1.386 (4)
C2—C3	1.528 (4)	C18—H18	0.9300
C2—H2A	0.9700	C19—H19	0.9300
C2—H2B	0.9700	C20—H20A	0.9600
C3—C4	1.524 (4)	C20—H20B	0.9600
C3—C28	1.527 (4)	C20—H20C	0.9600
C3—C27	1.534 (4)	C21—C22	1.381 (4)
C4—C5	1.499 (4)	C21—C26	1.384 (4)
C4—H4A	0.9700	C22—C23	1.391 (4)
C4—H4B	0.9700	C22—H22	0.9300
C5—C6	1.454 (4)	C23—C24	1.358 (5)
C6—C7	1.509 (4)	C23—H23	0.9300
C7—C8	1.501 (4)	C24—C25	1.344 (5)
C7—C21	1.528 (4)	C25—C26	1.378 (4)
C7—H7	0.9800	C25—H25	0.9300
C8—C13	1.349 (4)	C26—H26	0.9300
C8—C9	1.457 (4)	C27—H27A	0.9600
C9—C10	1.498 (4)	C27—H27B	0.9600
C10—C11	1.514 (5)	C27—H27C	0.9600
C10—H10A	0.9700	C28—H28A	0.9600
C10—H10B	0.9700	C28—H28B	0.9600
C11—C12	1.523 (4)	C28—H28C	0.9600
C11—C30	1.525 (5)	C29—H29A	0.9600
C11—C29	1.538 (5)	C29—H29B	0.9600
C12—C13	1.499 (4)	C29—H29C	0.9600
C12—H12A	0.9700	C30—H30A	0.9600
C12—H12B	0.9700	C30—H30B	0.9600
C14—C19	1.373 (4)	C30—H30C	0.9600
			
C1—N1—C13	119.5 (2)	C15—C14—N1	119.7 (3)
C1—N1—C14	120.3 (2)	C14—C15—C16	119.6 (3)
C13—N1—C14	119.2 (2)	C14—C15—H15	120.2
C6—C1—N1	121.0 (3)	C16—C15—H15	120.2
C6—C1—C2	122.2 (3)	C15—C16—C17	121.1 (3)
N1—C1—C2	116.8 (2)	C15—C16—H16	119.4
C1—C2—C3	113.8 (2)	C17—C16—H16	119.4
C1—C2—H2A	108.8	C18—C17—C16	118.2 (3)
C3—C2—H2A	108.8	C18—C17—C20	121.7 (3)
C1—C2—H2B	108.8	C16—C17—C20	120.1 (3)
C3—C2—H2B	108.8	C17—C18—C19	121.2 (3)
H2A—C2—H2B	107.7	C17—C18—H18	119.4
C4—C3—C28	109.9 (3)	C19—C18—H18	119.4
C4—C3—C2	107.7 (3)	C14—C19—C18	119.6 (3)
C28—C3—C2	110.8 (3)	C14—C19—H19	120.2
C4—C3—C27	109.9 (3)	C18—C19—H19	120.2
C28—C3—C27	109.4 (3)	C17—C20—H20A	109.5
C2—C3—C27	109.1 (3)	C17—C20—H20B	109.5
C5—C4—C3	114.0 (3)	H20A—C20—H20B	109.5
C5—C4—H4A	108.8	C17—C20—H20C	109.5
C3—C4—H4A	108.8	H20A—C20—H20C	109.5
C5—C4—H4B	108.8	H20B—C20—H20C	109.5
C3—C4—H4B	108.8	C22—C21—C26	118.3 (3)
H4A—C4—H4B	107.7	C22—C21—C7	121.3 (3)
O1—C5—C6	120.6 (3)	C26—C21—C7	120.4 (3)
O1—C5—C4	120.4 (3)	C21—C22—C23	120.5 (3)
C6—C5—C4	119.0 (3)	C21—C22—H22	119.7
C1—C6—C5	119.9 (3)	C23—C22—H22	119.7
C1—C6—C7	122.4 (3)	C24—C23—C22	118.5 (3)
C5—C6—C7	117.7 (2)	C24—C23—H23	120.8
C8—C7—C6	109.9 (2)	C22—C23—H23	120.8
C8—C7—C21	113.0 (2)	C25—C24—C23	122.8 (3)
C6—C7—C21	110.1 (2)	C25—C24—F1	119.1 (4)
C8—C7—H7	107.9	C23—C24—F1	118.1 (4)
C6—C7—H7	107.9	C24—C25—C26	118.7 (4)
C21—C7—H7	107.9	C24—C25—H25	120.6
C13—C8—C9	120.2 (3)	C26—C25—H25	120.6
C13—C8—C7	122.7 (3)	C25—C26—C21	121.2 (3)
C9—C8—C7	117.0 (2)	C25—C26—H26	119.4
O2—C9—C8	121.3 (3)	C21—C26—H26	119.4
O2—C9—C10	120.4 (3)	C3—C27—H27A	109.5
C8—C9—C10	118.2 (3)	C3—C27—H27B	109.5
C9—C10—C11	113.7 (3)	H27A—C27—H27B	109.5
C9—C10—H10A	108.8	C3—C27—H27C	109.5
C11—C10—H10A	108.8	H27A—C27—H27C	109.5
C9—C10—H10B	108.8	H27B—C27—H27C	109.5
C11—C10—H10B	108.8	C3—C28—H28A	109.5
H10A—C10—H10B	107.7	C3—C28—H28B	109.5
C10—C11—C12	108.8 (3)	H28A—C28—H28B	109.5
C10—C11—C30	110.3 (3)	C3—C28—H28C	109.5
C12—C11—C30	109.5 (3)	H28A—C28—H28C	109.5
C10—C11—C29	109.7 (3)	H28B—C28—H28C	109.5
C12—C11—C29	108.6 (3)	C11—C29—H29A	109.5
C30—C11—C29	110.0 (4)	C11—C29—H29B	109.5
C13—C12—C11	113.9 (3)	H29A—C29—H29B	109.5
C13—C12—H12A	108.8	C11—C29—H29C	109.5
C11—C12—H12A	108.8	H29A—C29—H29C	109.5
C13—C12—H12B	108.8	H29B—C29—H29C	109.5
C11—C12—H12B	108.8	C11—C30—H30A	109.5
H12A—C12—H12B	107.7	C11—C30—H30B	109.5
C8—C13—N1	120.7 (3)	H30A—C30—H30B	109.5
C8—C13—C12	122.8 (3)	C11—C30—H30C	109.5
N1—C13—C12	116.4 (2)	H30A—C30—H30C	109.5
C19—C14—C15	120.2 (3)	H30B—C30—H30C	109.5
C19—C14—N1	120.1 (3)		
			
C13—N1—C1—C6	9.2 (4)	C30—C11—C12—C13	−73.3 (4)
C14—N1—C1—C6	177.1 (3)	C29—C11—C12—C13	166.6 (3)
C13—N1—C1—C2	−170.4 (3)	C9—C8—C13—N1	174.7 (3)
C14—N1—C1—C2	−2.5 (4)	C7—C8—C13—N1	−2.9 (4)
C6—C1—C2—C3	21.9 (4)	C9—C8—C13—C12	−3.1 (5)
N1—C1—C2—C3	−158.5 (3)	C7—C8—C13—C12	179.4 (3)
C1—C2—C3—C4	−49.8 (3)	C1—N1—C13—C8	−11.6 (4)
C1—C2—C3—C28	70.4 (4)	C14—N1—C13—C8	−179.6 (3)
C1—C2—C3—C27	−169.1 (3)	C1—N1—C13—C12	166.3 (3)
C28—C3—C4—C5	−68.6 (3)	C14—N1—C13—C12	−1.7 (4)
C2—C3—C4—C5	52.1 (3)	C11—C12—C13—C8	−20.7 (5)
C27—C3—C4—C5	170.8 (3)	C11—C12—C13—N1	161.5 (3)
C3—C4—C5—O1	155.5 (3)	C1—N1—C14—C19	101.5 (3)
C3—C4—C5—C6	−26.6 (4)	C13—N1—C14—C19	−90.5 (3)
N1—C1—C6—C5	−172.3 (2)	C1—N1—C14—C15	−79.2 (4)
C2—C1—C6—C5	7.2 (4)	C13—N1—C14—C15	88.8 (3)
N1—C1—C6—C7	7.6 (4)	C19—C14—C15—C16	0.3 (5)
C2—C1—C6—C7	−172.8 (3)	N1—C14—C15—C16	−179.0 (3)
O1—C5—C6—C1	173.0 (3)	C14—C15—C16—C17	−0.2 (5)
C4—C5—C6—C1	−4.9 (4)	C15—C16—C17—C18	0.3 (5)
O1—C5—C6—C7	−6.9 (4)	C15—C16—C17—C20	−178.5 (3)
C4—C5—C6—C7	175.2 (3)	C16—C17—C18—C19	−0.6 (5)
C1—C6—C7—C8	−19.6 (4)	C20—C17—C18—C19	178.3 (3)
C5—C6—C7—C8	160.3 (2)	C15—C14—C19—C18	−0.5 (5)
C1—C6—C7—C21	105.5 (3)	N1—C14—C19—C18	178.8 (3)
C5—C6—C7—C21	−74.6 (3)	C17—C18—C19—C14	0.7 (5)
C6—C7—C8—C13	17.3 (4)	C8—C7—C21—C22	−110.1 (3)
C21—C7—C8—C13	−106.2 (3)	C6—C7—C21—C22	126.5 (3)
C6—C7—C8—C9	−160.4 (2)	C8—C7—C21—C26	70.8 (4)
C21—C7—C8—C9	76.2 (3)	C6—C7—C21—C26	−52.6 (4)
C13—C8—C9—O2	−179.3 (3)	C26—C21—C22—C23	0.8 (5)
C7—C8—C9—O2	−1.5 (4)	C7—C21—C22—C23	−178.3 (3)
C13—C8—C9—C10	−2.6 (5)	C21—C22—C23—C24	0.2 (5)
C7—C8—C9—C10	175.1 (3)	C22—C23—C24—C25	−1.1 (6)
O2—C9—C10—C11	−151.3 (3)	C22—C23—C24—F1	179.0 (3)
C8—C9—C10—C11	32.0 (4)	C23—C24—C25—C26	1.0 (6)
C9—C10—C11—C12	−53.0 (4)	F1—C24—C25—C26	−179.2 (3)
C9—C10—C11—C30	67.1 (4)	C24—C25—C26—C21	0.1 (6)
C9—C10—C11—C29	−171.6 (3)	C22—C21—C26—C25	−1.0 (5)
C10—C11—C12—C13	47.2 (4)	C7—C21—C26—C25	178.2 (3)

### Hydrogen-bond geometry (Å, °)

**Table:**

*D*—H···*A*	*D*—H	H···*A*	*D*···*A*	*D*—H···*A*
C15—H15···O1^i^	0.93	2.45	3.336 (3)	159

Symmetry codes: (i) −*x*+2, −*y*+1, −*z*.
